# Survey Result for E-labeling Initiatives in Asia

**DOI:** 10.1007/s43441-022-00462-5

**Published:** 2022-10-10

**Authors:** Rie Matsui, Koji Yamaguchi, Jadz Jevz Venzon Lee, Ivy Ting, Destita Khairilisani, Jenny Chang, Jeong-Min Seo, Ina Park, Alice Seat Mee Chee, Paul Marvin Quizon, Usanee Harnpramukkul, Ellen Sem, Thuy Nguyen, Anagha Padhye, Runyi Mo

**Affiliations:** 1 Pfizer R&D Japan, Tokyo, Japan; 2 Takeda Pharmaceutical Company Limited, Osaka, Japan; 3 Astellas Pharma Inc., Tokyo, Japan; 4 Ferring Pharmaceuticals Ltd., Kowloon, Hong Kong; 5 Abbott Indonesia, South Jakarta, Indonesia; 6 Merck Sharp & Dohme, Taipei, Taiwan; 7 KPBMA, Seoul, Korea; 8 GE Healthcare AS Korea, Seoul, Korea; 9 Pharmaceutical Association of Malaysia (PhAMA), Selangor, Malaysia; 10 Pfizer, Inc. (Philippines), Makati, Philippines; 11 Pfizer (Thailand) Limited, Bangkok, Thailand; 12 Present Address: Takeda Pharmaceuticals International AG, Singapore, Singapore; 13 Johnson & Johnson Pte Ltd, Singapore, Singapore; 14 Pharma Group Vietnam, Ho Chi Minh, Vietnam; 15 Sanofi GRSP China Team, Beijing, China; 16 Pfizer Limited (India), Mumbai, India

**Keywords:** e-Labeling, Electronic product information, Asia, APAC, Survey

## Abstract

Under the COVID-19 pandemic, various electronic labeling initiatives have accelerated worldwide in the healthcare and pharmaceutical fields as part of a wider digital transformation [1, 2]. Although there is no universal definition of electronic labeling (e-labeling) globally, it is widely understood that e-labeling refers to the product information that is distributed via electronic means. There are 5 factors to be considered in e-labeling, and these are discussed in this publication. APAC is an industry-driven initiative with 13 R&D-based pharmaceutical associations joining from 11 markets in Asia. e-labeling was discussed as a new topic starting in 2020, and a 22-question survey was conducted in November 2021 to understand the current e-labeling status. The survey results showed that e-labeling initiatives were at different levels of maturity in the Asian region, although most markets have started to discuss e-labeling initiatives. Various challenges exist around e-labeling initiatives due to a variety of different approaches being taken in the region. It would be advisable to develop regional guidance on how to proceed with e-labeling initiatives in the Asian region to have a consistent and efficient approach. The close collaboration between agencies, Health Care Professionals (HCPs), patients, and industry associations is important to move e-labeling initiatives forward in Asia.

## Introduction

### Role of Labeling

Labeling is a key component of the application dossier in the Common Technical Document (CTD) for new marketing authorizations. Labeling is one of the most important official documents and important in all stages of the product lifecycle as it provides essential product information in terms of safety, efficacy, and quality in a timely manner. There are a variety of formats (e.g., paper, electronic) and types (e.g., for patients or HCP) distributed according to different national requirements. The labeling is a critical risk minimization measure as from time to time, and there could be new safety or efficacy information about the product, either from post-marketing surveillance or new clinical studies (e.g., new indications or formulations) or risk concerns which should be reflected in the product labeling [3, 4]. There are two types of labeling: HCP and Patient labeling. HCP labeling where required by local regulation, is the medical summation of product information intended for use by HCPs and usually contains more comprehensive information to describe the benefit-risk profile. Patient labeling/Patient Information Leaflet (PIL) is primarily intended for patients and is written in patient-friendly language [5–7].

In most markets, it is mandated by regulation to insert the product label in the commercial pack. Depending on the market, the pack may contain healthcare professional information (e.g., United States, Japan) and/or patient information (e.g., European Union).

## What is e-Labeling?

Under the COVID-19 pandemic, various e-labeling initiatives have begun worldwide in the healthcare and pharmaceutical fields as part of a wider digital transformation.　This is because of the need for immediately available updated labeling, which can be achieved by adopting dynamic e-labeling procedures to ensure that the most updated labeling is made available and accessible to different stakeholders [8, 9]. Through these initiatives, the regulators, industry, HCPs, and patients understand that e-labeling will deliver the most updated labeling to HCPs and patients and will improve the accessibility and understanding of approved medical product information, thereby enhancing adherence to medicines and improving patient outcomes [1, 10]. E-labeling also provides an opportunity to deliver personalized [11], user-friendly information and enhanced features for patients with visual and hearing disabilities. Although there is no universal definition of e-labeling globally, it is widely accepted that e-labeling refers to the product information that is distributed via electronic means.

There are 5 factors to be considered in e-labeling:

(1) availability of the latest labeling on a publicly accessible website (e.g., product information available online);

(2) accessible, reader-friendly format (e.g., scanning a machine-readable code);

(3) eliminating paper labeling from commercial pack;

(4) common electronic standard (e.g., structured content); and

(5) efficient information flow (e.g., interoperability between systems).

The availability of the latest labeling on a publicly accessible website is an important first step in improving patient safety and trust in medicines [1, 10]. The adoption of e-labeling with improved accessibility will enhance the user’s experience to navigate around the product information in a more user-friendly manner, and to better understand correct usage as well as the safety and efficacy profile of the drug. Eventual transformation from paper labeling to e-labeling will shorten the lead time to launch new products, improve efficiencies by eliminating the operational steps of inserting paper labeling in packs, and support environmental-friendly practices. In the future, e-labeling can be integrated with the wider digital healthcare system such as electronic medical records, resulting in greater efficiencies, and opportunities across a wide spectrum within healthcare.

In the Asian region, Japan [12, 13] and Singapore [14] have implemented e-labeling, and discussions on e-labeling initiatives have also been started in several markets (e.g., Taiwan [15], Thailand).

The purpose of the survey is to understand the current status of e-labeling across the 12 markets in the Asian region and confirm needs for industry associations. In addition, the survey aims to determine current challenges and possible approaches to be utilized in order to move forward with e-labeling initiatives in the Asian region.

## Asia Partnership Conference of Pharmaceutical Associations (APAC)

APAC is an industry-driven initiative with thirteen R&D-based pharmaceutical associations joining from eleven markets in Asia to work together to realize the mission, “To expedite the launch of innovative medicines for the people in Asia.” Since its establishment in 2012, APAC has provided unique opportunities for constructive discussions among regulators, academia and industry on timely topics to improve “Access to Innovative Medicines” (ATIM) in Asia. APAC has begun discussing E-labeling as a new topic in 2020 and an APAC e-labeling Expert Working Group (EWG) was established in 2021 (See Table 1)

**Table 1:** Asia Partnership Conference of Pharmaceutical Associations

HKAPI	The Hong Kong Association of the Pharmaceutical Industry
IPMG	International Pharmaceutical Manufacturers Group
IRPMA	International Research-based Pharmaceutical Manufacturers Association
JPMA	Japan Pharmaceutical Manufacturers Association
KPBMA	Korea Pharmaceutical and Bio-Pharma Manufacturers Association
KRPIA	Korean Research-based Pharmaceutical Industry Association
OPPI	Organization of Pharmaceutical Producers of India
PhAMA	Pharmaceutical Association of Malaysia
PHAP	Pharmaceutical and Healthcare Association of the Philippines
PhIRDA*	China Pharmaceutical Innovation & Research Development Association
PReMA	Pharmaceutical Research & Manufacturers Association
RDPAC	R&D-based Pharmaceutical Association Committee
SAPI	Singapore Association of Pharmaceutical Industries

^*^PhIRDA is not a member of the APAC e-labeling EWG and did not join the survey

.

## Methods

A 22-question survey was completed by APAC’s 12* member associations and Pharma Group Vietnam in November 2021 to understand the current e-labeling status in 12 markets in the Asian region.​ Each member association made only one response to the survey. There were 4 questions related to the general labeling requirements and 18 questions to inquire about the current status of e-labeling initiatives. The scope of the survey is for all prescription drugs, and over-the-counter (OTC) drugs is out of scope.

* One member association from each market completed the survey except for Korea, where 2 member associations completed it. For Korea, responses are consolidated and counted as one response if the questions are related to regulation or pharmaceutical law.

## Results

### General Labeling Requirements

**Figure 1:**
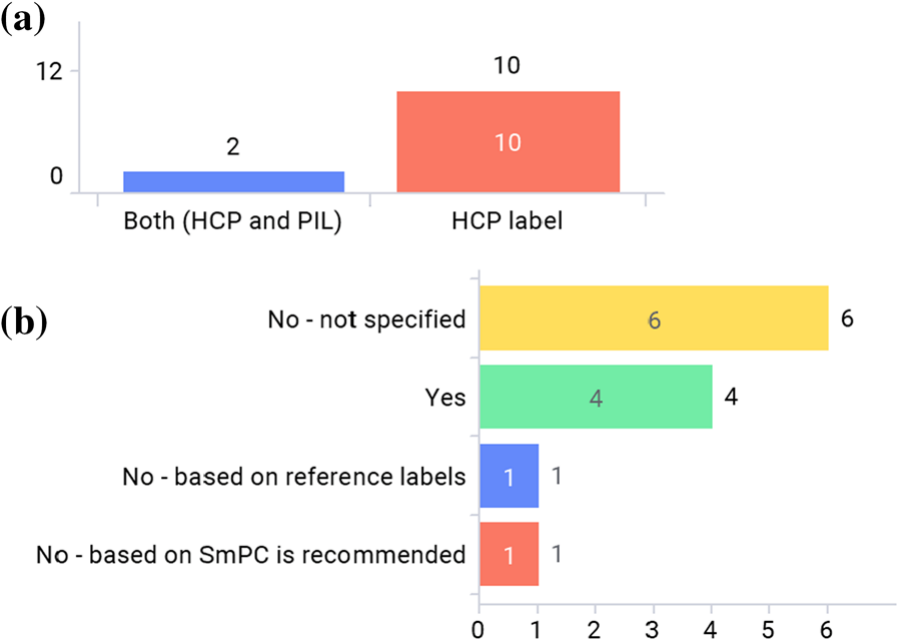
**a** What paper-package insert is in the commercial pack? **b** Is there a mandatory national single template for HCP labeling that is implemented in your market?

Figure 1a shows what paper-package inserts are found in the commercial pack within the Asian region. Only 2 markets—Thailand [16] and Indonesia [17]—have both HCP label and PIL in the commercial pack. Figure 1b shows that more than half of markets do not have a mandatory national single template for HCP labeling, which means the ordering of the sections and section names are different depending on the product labeling even within the same market. In situations like this, e-labeling can significantly improve searchability.

**Figure 2:**
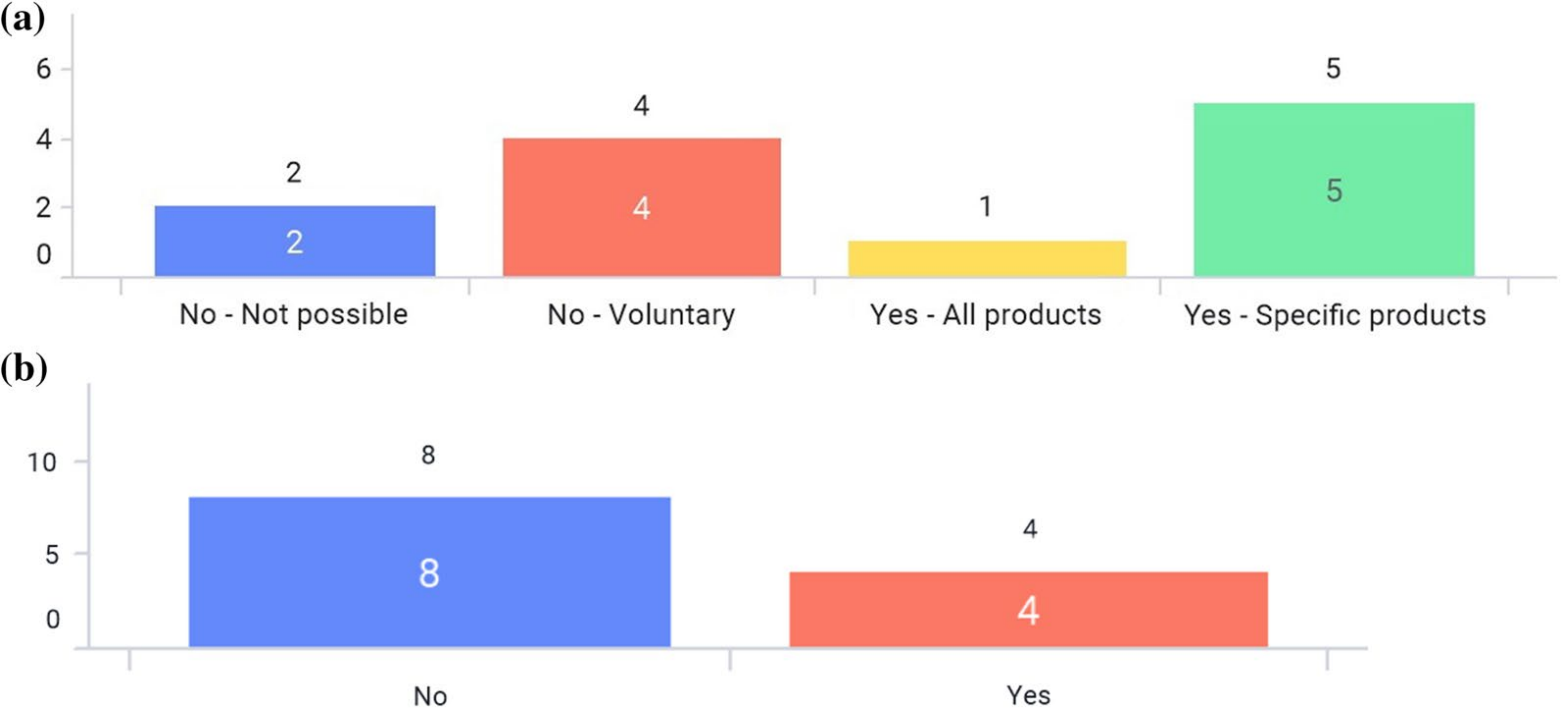
**a** Is the Patient Information Leaflet (PIL) for prescription drugs required by regulations or laws? **b** Is there a mandatory national single template for PIL for prescription drugs that is implemented in your market?

**Figure 3:**
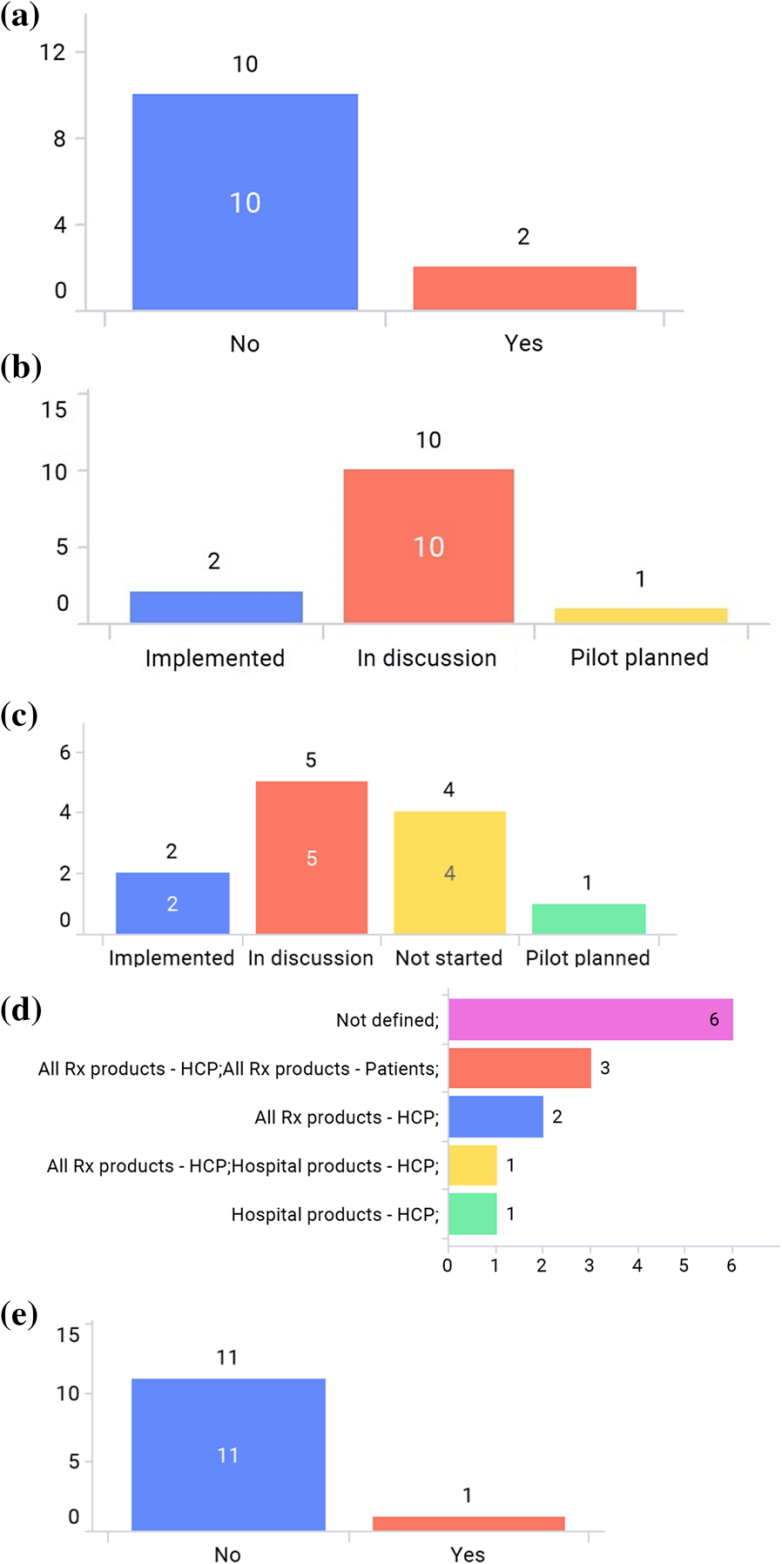
**a** Are there any e-labeling-related guidance? **b** Has your association raised e-labeling topics or started discussion on e-labeling? **c** Has your market’s HA (Health Authority) raised e-labeling topics or started discussion on e-labeling? **d** Which products are considered for e-labeling? (Multiple answers allowed) **e** Is there any back-up for e-labeling, such as a call center?

**Figure 4:**
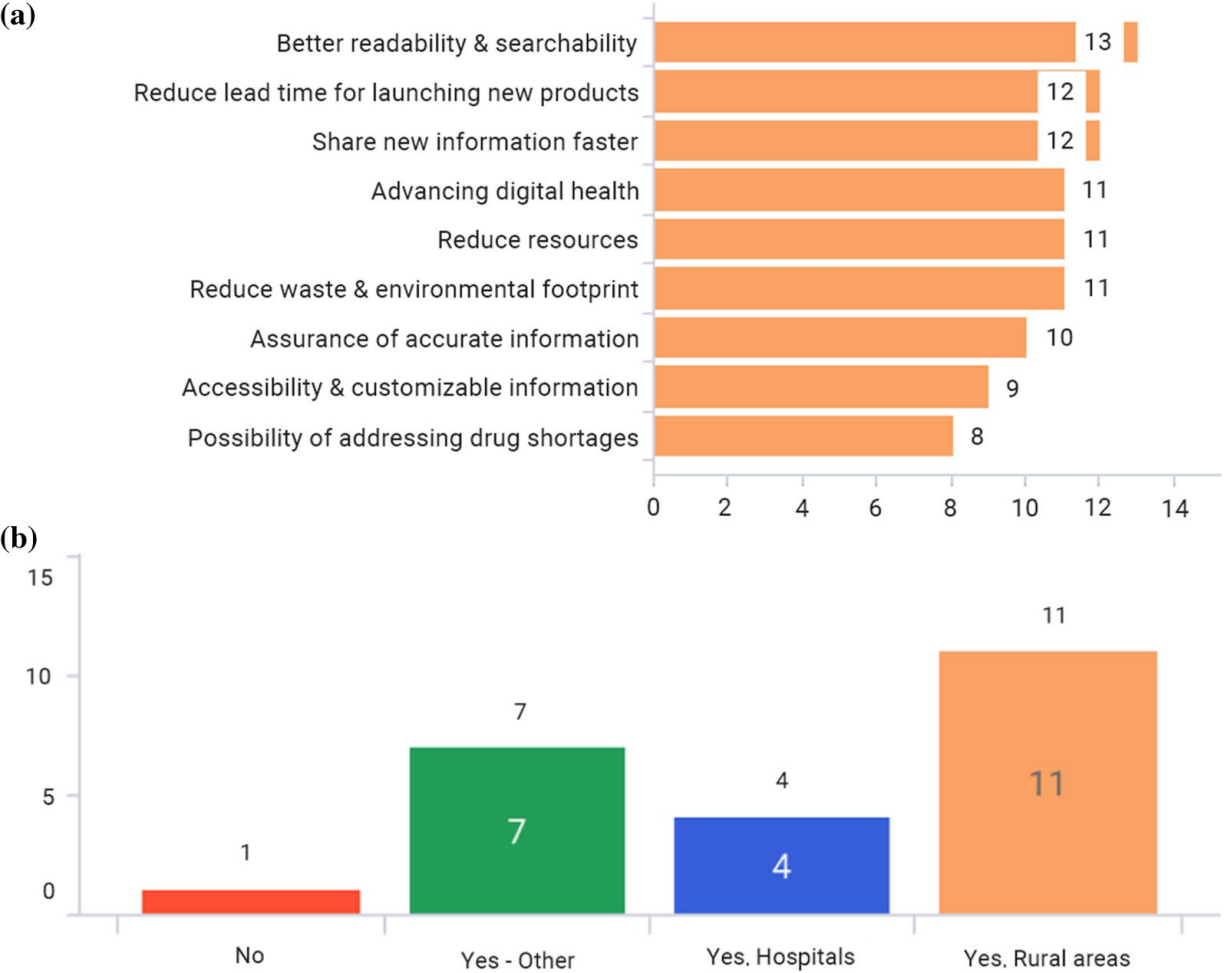
**a** Which of the following benefits would you like to achieve with the implementation of e-labeling? (Multiple answers allowed) **b** Are there any concerns about internet access to implementing e-labeling? (Multiple answers allowed)

As shown in Fig. 2a, the PIL for most prescription drugs is not required by regulations or laws in the majority of the markets, since only Indonesia requires the PIL for all products [17]. Figure 2b shows that more than two thirds of markets do not have a mandatory national single template for the PIL.

## Current Status of E-Labeling Initiatives

### Overall Progress

Only 2 markets—Japan and Singapore—have issued e-labeling related guidance in Asia (Fig. 3a). For Japan, the regulatory requirement to enclose paper-package insert to an individual product container (package) of medicinal products has been enforced on Aug 1st, 2021 (with a 2-year grace period before it goes into full effect). In this implementation, there are three major changes in operation. First, paper-package insert will no longer be enclosed in product containers (packages), and package inserts are viewed electronically on the Pharmaceuticals and Medical Devices Agency (PMDA) website. Second, the GS1 symbol including the GS1 product code is required to be displayed on the product container (package). Reading software is distributed to link the GS1 code to the package insert posted on the PMDA website. Package insert information (cautionary information etc. to consider) posted on the PMDA website is regarded as formal information. Third, the establishment of an organization/system to provide package insert information is required [12, 13].

In the Singapore guidance published on 30 April 2021, the document describes e-labeling is applicable to prescription-only medicines only, and Marketing Authoring Holders (MAHs) are responsible for ensuring that the e-labels published on its websites are aligned to the most up-to-date product information within 30 days after the approval by Health Sciences Authority (HSA). MAHs should state the Uniform Resource Locator (URL) (short link preferred), QR code, or other machine-readable code on the outer packaging. Also, it mentions e-labeling should be accessible to all users or catered to targeted users. It is voluntary for MAHs to implement e-Labeling where physical labels need not be distributed with the pack [14]. All associations have implemented, planned pilots, or discussed e-labeling (Fig. 3b). More than half of the markets have started to discuss e-labeling or planned pilot programs with their respective regulatory authorities. (Fig. 3c). Figure 3d shows that prescription drug and/or hospital products are considered in scope for e-labeling as answered by most associations. One regulatory agency—the Japan PMDA—has a back-up platform for e-labeling in case the server is offline as the electronic labels are hosted on the PMDA’s website (Fig. 3e).

Figure 4a shows that all associations believe that there are a number of benefits that can be achieved with the implementation of e-labeling, listing better readability and searchability for users as the most important benefit. On the other hand, Fig. 4b shows that there are some concerns about internet access to implement e-labeling in certain rural areas in the majority of markets. However, internet accessibility in rural areas may improve over time due to better infrastructure, or there could be dedicated hospitals or pharmacies equipped with better internet accessibility to address this issue [18].

The reduction in the volume of paper-package inserts for HCPs in commercial packs can be estimated based on the drug price and annual sales of manufactured drugs in concerned markets. For example in Japan, it is estimated by the authors that about 10 billion paper-package inserts can be reduced in Japan, given that 1 paper per 1 manufactured product is produced [19, 20]. The impact in the wider Asian region could be as high as 40 billion (calculated by the author) [21] if every package insert is replaced with e-labeling for information distribution.

## (1) Availability of the Latest Labeling on a Publicly Accessible Website (Product Information Available Online)

**Figure 5:**
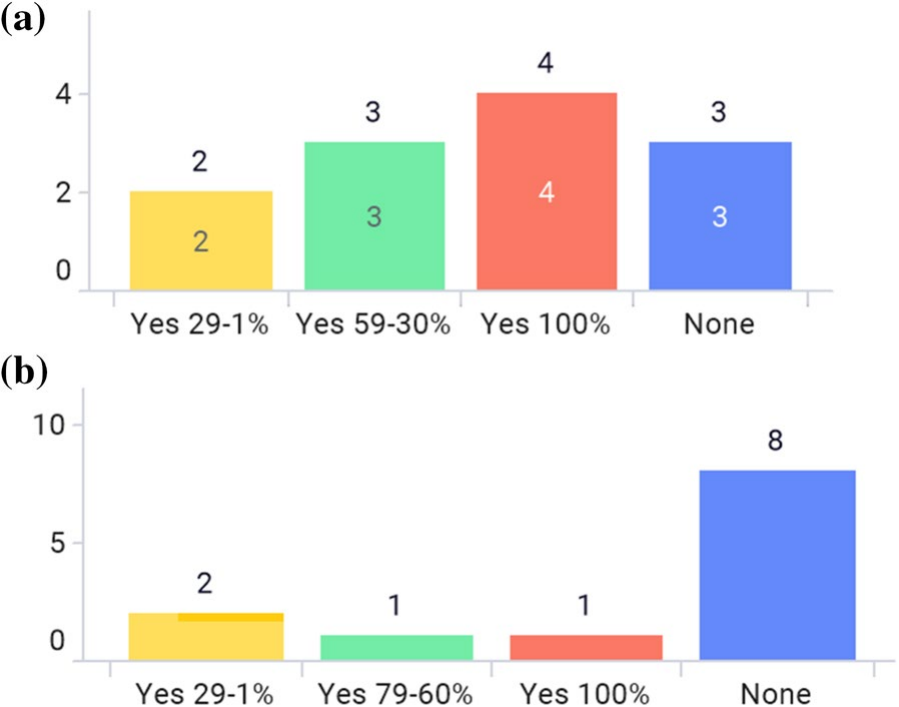
**a** Is the HCP labeling information available on the HA website? **b** Is the patient labeling information available on the HA website?

Figure 5a and b shows the availability of HCP labeling information and patient labeling information, respectively, on Health Authority (HA) websites. 4 markets have published HCP labels for all products* on HA websites centrally and 5 markets have published them partially as well. 3 markets have not published labels on the HA website. On the other hand, only 4 markets have published patient labels on the HA website while 8 markets have not published patient labels on the HA website.

* One market publishes innovator product labeling only.

## (2) Accessible, Reader-Friendly Format, e.g., Scanning a Code

**Figure 6:**
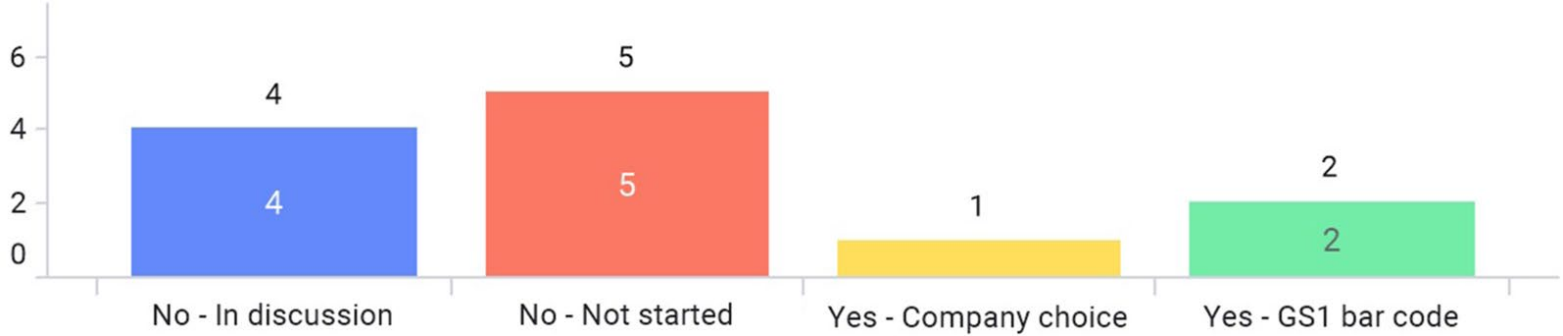
Has any accessibility measure been taken to connect e-labeling information with the packaging as implemented by a regulation in your market?

Only 2 markets—Japan and Taiwan—have started to use GS1 barcodes to connect e-labeling information with the packaging. In Singapore, this accessibility measure is dependent on company choice. 4 markets are currently under discussion in connecting e-labeling information with the packaging. But, almost half of the markets have not started to discuss yet (Fig. 6).

**Figure 7:**
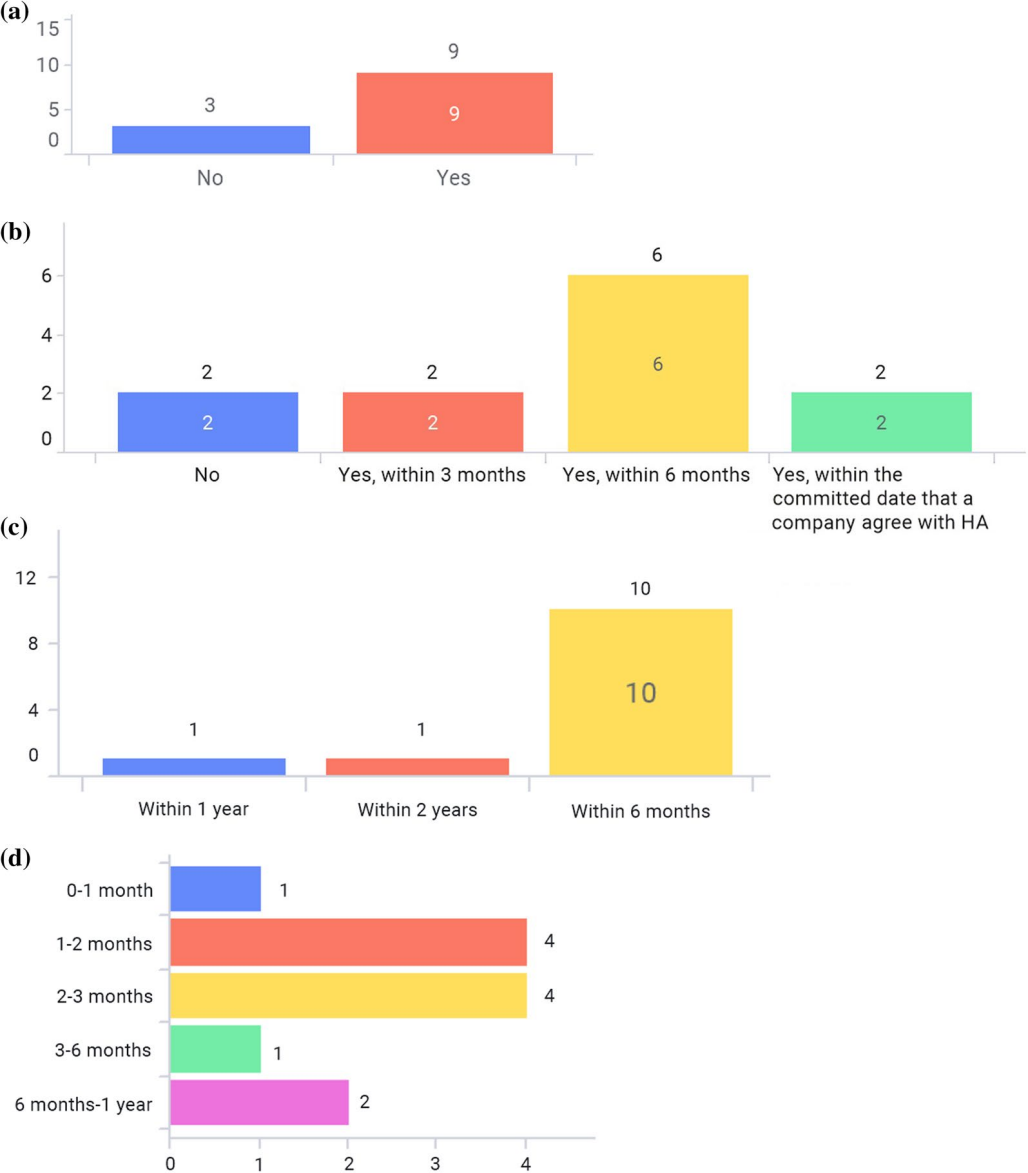
**a** Is the paper-package Insert (labeling) in commercial pack required by regulations or laws? **b** Are there any regulations for timeline from approval of labeling revision until commercial products with updated labeling artwork in the market? **c** How long does it take to implement an updated artwork for labeling revision from artwork development initiation to batch release? (Average) **d** If e-labeling is implemented with operation removal of inserting paper labeling into the commercial pack, how much lead time reduction can you expect to launch new products?

## (3) Eliminating Paper Labeling from Commercial Pack

As illustrated in Fig. 7a, the majority of markets still require paper-package inserts (labeling) for HCPs in the commercial pack as required by national regulations or laws. In order to eliminate paper labeling from commercial packs as a result of a requirement from a particular market, revision of the regulations or laws are needed to be implemented first. According to the e-labeling guidance/regulation, 2 markets—Japan and Singapore—can officially remove paper labeling from the commercial pack. In Thailand, the local law [16] can be interpreted as accepting either paper or paperless package inserts in commercial packs. However, there still remain other challenges to remove paper/printed package insert locally, such as internet access in rural areas, and patients/consumers’ behaviors at this moment to accept e-labeling.

Figure 7b shows that there is a timeline requirement from approval of labeling revision until the release of commercial products with updated labeling artwork in the majority of the markets. Figure 7c shows that most markets are required to update labeling artwork in commercial products within 6 months after labeling revision approval. Figure 7d shows how much e-labeling can reduce the lead time in launching new products if there is no need to insert paper labeling into commercial packs. If e-labeling is implemented, the most updated labeling information will be delivered in a timely manner without worrying about the artwork requirement timeline and, thus, contribute to a more stable, predictable supply.

## (4) Common Electronic Standard, e.g., Structured Contents

**Figure 8:**
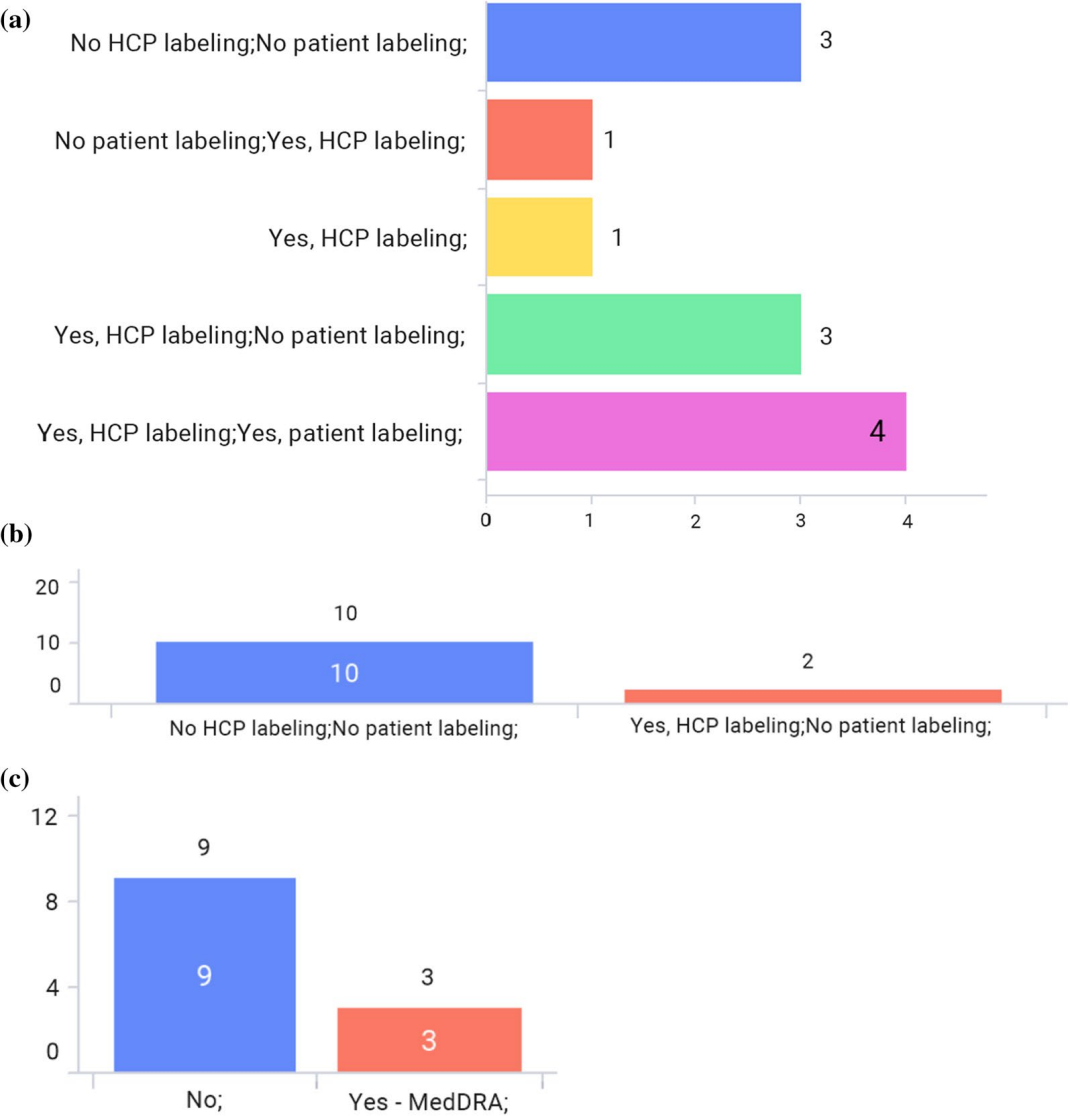
**a** Is the labeling information available in PDF format on the HA and/or third-party website? **b** Is the labeling information available in XML on the HA and/or third-party website? **c** Are there any standardized dictionaries relevant for labeling (such as MedDRA, SNOMED, UCUM, EDQM, SPOR) that are required/recommended at the national level? (Multiple answers allowed)

Figure 8a shows that in the majority of markets, labels are published in a PDF format. Figure 8b shows that the labeling information is available in Extensible Markup Language (XML) on the HA website for only 2 markets—Japan and Korea. Figure 8c shows that no other standardized dictionaries are used except for MedDRA.

## (5) Efficient Information Flow, e.g., Interoperability Between Systems

**Figure 9:**
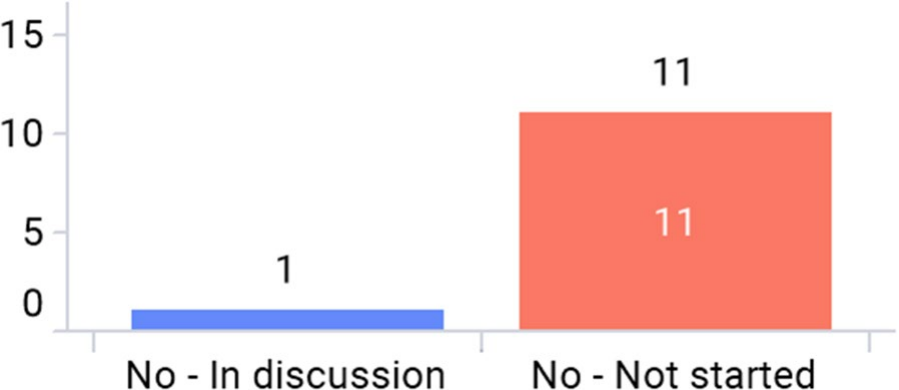
Is any of interoperability standards used in labeling database on the HA or third-party website?

No interoperability for e-labeling has been implemented and even the discussion has not been started yet (Fig. 9).

## Discussion

The COVID-19 pandemic accelerated the use of technology to enhance access to information. Product labeling, which has traditionally been printed and formed part of the packaging, is now slowly being made accessible electronically. Publishing labeling is already being implemented in other parts of the world [22–24]. While some markets participating in this survey have laid the foundations for e-labeling pre-pandemic, there is no doubt that the COVID-19 facilitated its implementation.

As the e-labeling system is relatively new in some markets and others are yet to begin on its implementation, challenges have been identified. For COVID-19 vaccines, instead of inserting paper labeling, e-labeling has been accepted to improve regulatory agility as an exceptional case [2]. e-labeling meets the challenges of the pandemic and is a critical method to deliver vaccines/medicines quickly and globally. Labels have been published on the central database in many markets, but in reality, they are often not updated in a timely manner. One key area is to establish a robust process to publish updated labeling in a timely manner in the database. The centralized database should be a “single source of truth” for HCPs and patients which can improve trust and credibility. PIL for many products may not be available in the Asian region. In order for patients to understand the product information, the PIL should be written in patient-friendly language. Thus, it would be necessary to discuss the availability of patient-friendly product information. As pdf format is still used commonly in the industry. To maximize the full benefits of e-labeling, a structured content format will enable easy searching, tracking, reuse, and integration with other digital healthcare platforms. Some markets in the Asian region have implemented or started a pilot program with putting QR code or GS1 bar code/ two-dimensional (2D) codes [25] on carton, which can be scanned via mobile device and linked to product information in an electronic way.

Since there are concerns about internet connectivity in rural areas [18], and emerging risks in the internet such as cybersecurity, a step-by-step approach to improve digital infrastructure may need to be considered in order to progress e-labeling initiatives. In the majority of markets, a paper-package insert in the commercial pack is still required, and the timeline of artwork implementation for a paper-package insert is set as within 6 months. With paper-package insert, it can be delivered anywhere physically and securely. However, it requires more time for delivery as compared with e-labeling. If paper labeling can be eliminated from the commercial pack in the future after reducing concerns around internet connectivity, it will improve efficiency by eliminating operational steps of inserting paper labeling in packs and support environmental-friendly practices. However, packaging specifications might have an impact in implementing e-labeling practices. The risk assessment to eliminate paper labeling from the commercial pack should be made, since the paper label may act as a cushion material [10]. The step-wise approach such as keeping a paper labeling after implementing e-labeling for a period of time would be an option, so that all sides including the regulators, HCPs and patients, would be comfortable with the transition to e-labeling. For the markets that have a mandate to insert paper labeling, it would be preferable to start discussing advocacy plans to eliminate paper labeling from the commercial pack in appropriate timing and its process, such as implementing a step-wise approach, to engage between regulatory authority and industry associations in order to move forward e-labeling discussions.

## Conclusion

**Figure 10:**
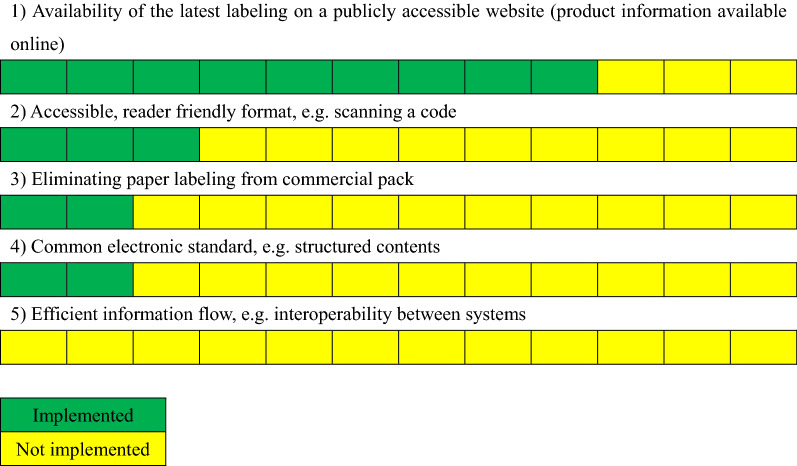
Status of e-labeling implementation

As the e-labeling survey shown in Fig. 10, there are different levels of e-labeling initiatives in the Asian region, although most of the markets have started to discuss e-labeling initiatives. The discussion on e-labeling initiatives is still at early stage in the majority of markets. There is a challenge to e-labeling initiatives due to a variety of approaches in the region. In that case, companies have to follow each market’s approach, although the manufacturing sites have been integrated across regions. It will be difficult and inefficient, and this will bring a negative impact on the implementation on e-labeling. Therefore, it would be advisable to develop a regional guidance on how to proceed e-labeling initiatives in the Asian region to have a consistent approach. Asian counties can collaborate further in terms of e-labeling although the guidance will neither be intended to mandate nor to include the implementation plan for each market. The close collaboration between agencies, HCPs, patients, and industry associations is important to move the e-labeling initiative forward in Asia.
