# Publisher Correction: Survey Result for E-labeling Initiatives in Asia

**DOI:** 10.1007/s43441-022-00476-z

**Published:** 2022-10-31

**Authors:** Rie Matsui, Koji Yamaguchi, Jadz Jevz Venzon Lee, Ivy Ting, Destita Khairilisani, Jenny Chang, Jeong-Min Seo, Ina Park, Alice Seat Mee Chee, Paul Marvin Quizon, Usanee Harnpramukkul, Ellen Sem, Thuy Nguyen, Anagha Padhye, Runyi Mo

**Affiliations:** 1 Pfizer R&D Japan, Tokyo, Japan; 2 Takeda Pharmaceutical Company Limited, Osaka, Japan; 3 Astellas Pharma Inc., Tokyo, Japan; 4 Ferring Pharmaceuticals Ltd., Kowloon, Hong Kong; 5 Abbott Indonesia, South Jakarta, Indonesia; 6 Merck Sharp & Dohme, Taipei, Taiwan; 7 KPBMA, Seoul, Korea; 8 GE Healthcare AS Korea, Seoul, Korea; 9 Pharmaceutical Association of Malaysia (PhAMA), Selangor, Malaysia; 10 Pfizer, Inc. (Philippines), Makati, Philippines; 11 Pfizer (Thailand) Limited, Bangkok, Thailand; 12 Takeda Pharmaceuticals International AG, Singapore, Singapore; 13 Johnson & Johnson Pte Ltd, Singapore, Singapore; 14 Pharma Group Vietnam, Ho Chi Minh, Vietnam; 15 Pfizer Limited (India), Mumbai, India; 16 Sanofi Global Regulatory Science & Policy, China & Asia team, Beijing, China

## Correction to: Therapeutic Innovation & Regulatory Science https://doi.org/10.1007/s43441-022-00462-5

Due to a Production error the references were numbered incorrectly. Reference 14, "Singapore", was split into two references (14 and 15). So, reference 16 "Taiwan", should be renumbered as "15", reference 17, "Thailand", should be renumbered as "16", and so on through the rest of the references (the reference number callouts within the text of the article are correct).

